# Staying ‘Covid‐safe’: Proposals for embedding behaviours that protect against Covid‐19 transmission in the UK

**DOI:** 10.1111/bjhp.12557

**Published:** 2021-08-31

**Authors:** Susan Michie, Robert West, Nick Pidgeon, Stephen Reicher, Richard Amlôt, Laura Bear

**Affiliations:** ^1^ Centre for Behaviour Change University College London UK; ^2^ Institute of Epidemiology and Health University College London UK; ^3^ Understanding Risk Research Group School of Psychology Cardiff University UK; ^4^ School of Psychology and Neuroscience University of St. Andrews UK; ^5^ Behavioural Science and Insights Unit Public Health England UK; ^6^ Department of Social Anthropology London School of Economics and Political Science UK

**Keywords:** behaviour change, Covid‐19, long‐term, maintaining, policy, SARS‐CoV‐2, sustaining

## Abstract

**Objectives:**

The Scientific Pandemic Insights group on Behaviours (SPI‐B) as part of England’s Scientific Advisory Group on Emergencies (SAGE), were commissioned by the UK Cabinet Office to identify strategies to embed infection control behaviours to minimize Covid‐19 transmission in the long term.

**Methods:**

With minimal direct evidence available, three sources of information were used to develop a set of proposals: (1) a scoping review of literature on sustaining behaviour change, (2) a review of key principles used in risk and safety management, and (3) prior reports and reviews on behaviour change from SPI‐B. The information was collated and refined through discussion with SPI‐B and SAGE colleagues to finalize the proposals.

**Results:**

Embedding infection control behaviours in the long‐term will require changes to the financial, social, and physical infrastructure so that people in all sections of society have the capability, opportunity, and motivation needed to underpin those behaviours. This will involve building Covid‐safe educational programmes, regulating to ensure minimum standards of safety in public spaces and workspaces, using communications and social marketing to develop a Covid‐safe culture and identity, and providing resources so that all sections of society can build Covid‐safe behaviours into their daily lives.

**Conclusions:**

Embedding ‘Covid‐safe’ behaviours into people’s everyday routines will require a co‐ordinated programme to shape the financial, physical, and social infrastructure in the United Kingdom. Education, regulation, communications, and social marketing, and provision of resources will be required to ensure that all sections of society have the capability, opportunity, and motivation to enact the behaviours long term.

**Statement of contribution**

***What is already known about this subject?***

- Behaviour change requires capability, opportunity and motivation
- Maintaining health‐protective behaviours in the long‐term is difficult
- Establishing new identities, routines and norms, and providing support can be effective

***What does this study add?***

- Applying broad behaviour change principles may help sustain Covid‐protective behaviours
- A risk‐management approach addressing capability, opportunity and motivation is needed
- This will involve education, regulation, social marketing, and provision of resources

## Background

The global experience of 2020–2021 has demonstrated that it is highly likely that COVID‐19 will become endemic, with an ongoing potential for outbreaks resulting in part from new variants. As legal restrictions on social contact are eased, maintaining low levels of transmission will require policies that promote Covid‐protective behaviours in appropriate settings and at appropriate times (UK Government, 2021a; UK Government, 2021b, p. 76; UK Government, 2021c; UK Government, 2021d; UK Government, 2021e; UK Government, 2021f; UK Government, 2021g, p. 85). This is in addition to improved public health measures such as case finding, supported isolation, and border controls when necessary. The everyday behaviours include, when there is an elevated risk, some level of social distancing, wearing face coverings in enclosed spaces, and ensuring that indoor spaces are adequately ventilated. These protective behaviours are in addition to ongoing adherence to good general infection prevention and control behaviours (especially when symptomatic) such as respiratory and hand hygiene, and routine cleaning of commonly touched surfaces, such as door handles. They take place across multiple settings, including homes, public spaces, educational facilities, businesses, and hospitality and leisure facilities. This paper describes the methods and results of government advisory group’s attempt to develop proposals to embed these behaviours in the UK population, with a focus on (1) maintaining physical distance, (2) wearing face coverings, (3) ensuring adequate ventilation, and (4) working from home.

Changing and maintaining behaviour in the long‐term can be challenging, as evidenced by systemic reviews of evidence, for example those in the Cochrane Library (Cochrane Reviews, 2021). The evidence suggests that the most effective interventions are those that are multi‐faceted, co‐ordinated, and sustained, and aim to increase motivation, capabilities, and/or opportunities for behaviour change (Abraham, Kelly, West, & Michie, 2009; National Institute for Health & Care Excellence, 2007, 2014).

Many countries have multidisciplinary scientific groups advising Governments’ pandemic responses and policies. In the United Kingdom, this involves a strong behavioural science group that is tasked with providing advice on a wide variety of questions (UK Government, 2021g). One question concerned how to sustain infection control behaviours in the long‐term to keep rates of Covid‐19 transmission, illness, and death to a low level after legal restrictions were lifted. The paper summarizes the methods used to rapidly review relevant evidence and principles and the resulting proposals for the UK Government.

## Methods

A multidisciplinary group of behavioural and social scientists advising the UK Government on COVID‐19 contributed to the analysis and writing of advice through the Government Office for Science, led by authors SM and RW. A tight timescale for meeting a deadline set by the UK Government’s Cabinet Office meant that evidence gathering and synthesis had to be rapid. Literature from three sources was reviewed using rapid review methods (Gale et al., 2019) and the findings categorized according to the COM‐B model (West & Michie, 2020). This formed the basis for discussion and agreed proposals by a group established for this purpose by the Scientific Pandemic Insights Group on Behaviours (SPI‐B).

### Scoping review of the research literature on sustained behaviour change

We reviewed evidence on sustaining behaviours using the search terms ‘sustained behaviour change’ and ‘sustained social practice’ in Google Scholar and built a list of behaviour change principles until no new principles were identified using this method. The reason for using this search engine is that it covers reports from think tanks and government agencies as well as the mainstream academic literature and it sorts the results using an algorithm that prioritizes relevance and usage.

The total number of hits for the term ‘sustained behaviour change’ was approximately 1 million but as with any Google scholar search it would be expected that the large majority of these would have very low relevance. The search results were ordered by what the Google algorithm considered most to least relevant. They included journal articles, books, book chapters, letters, and reports. Documents were read in the order of search results to extract principles that had been successfully applied in a given context for a given behaviour or set of behaviours. The aim was to build an inclusive list of such principles. For each new document, the principles were matched to ones already in the list. If it was not there, it was added to the list and the behaviour or domain of interest was recorded. The search was terminated when no new principles had been extracted after five new documents had been read. The principles were classified according to whether they primarily addressed issues of capability, opportunity, or motivation.

### Analysis of the research literature on risk and safety management

We examined the risk and safety management literature for: (1) principles relevant to Covid‐19 protective behaviours and for which there is broad agreement in the field, and (2) examples of how behaviour has been changed in other settings relevant to sustaining COVID‐protective behaviours. This literature is potentially very wide, covering specialist Journals such as Risk Analysis or Safety Sciences, more general peer‐reviewed sources which reference the topic of risk, as well as multiple government and other publications for guidance on managing risk. This section is the collective product of a number of targeted review actions undertaken to identify best practice in risk assessment and its management, conducted primarily by the 3rd author (Pidgeon) who is a specialist in human behaviour and risk research, as part of science advice given to United Kingdom and Wales governments science at various points over the preceding 12 months. Hence, it involved a degree of expert judgement in collecting together and synthesizing all of these evidence‐based core principles, and in particular presenting only ones which are commonly agreed in the scientific literature, together with the (admittedly scarce) scientific evidence from other pandemics alongside the emerging studies of responses to Covid‐19.

### Analysis of the social psychological literature on social influence and norm formation

We examined the social psychological literature for (1) principles relevant to the collective understanding of new phenomena, specifically new scientific phenomena, (2) material relevant to how collective behaviours can be sustained and/or changed, and (3) examples of how normative processes have been used to change behaviour.

### Review of reports from the UK Government’s behavioural subgroup of the Scientific Advisory Group on Emergencies (SAGE)

We reviewed published SPI‐B reports to identify principles and evidence relating to sustained behaviour change.

### Expert consensus

The principles generated by the reviews were discussed by the authorship team and organized according to the capability‐opportunity‐motivation‐behaviour (COM‐B) model, a simple and actionable framework for understanding behaviour and developing behaviour change interventions by national government and other sectors (West, Michie, Chadwiock, Atkins, & Lorencatto, 2020). Thus, the principles were identified as targeting primarily people’s capability (e.g., knowledge and skills), opportunity (e.g., provision of resources and shaping of social norms), or motivation (e.g., targeting emotional responses or retraining habits). For each principle, the source in the review was identified together with the behavioural domain to which it had been applied. We examined evidence on inequalities as they relate to these behavioural domains in the light of the above and developed a set of principles for addressing these. The principles were collated and discussed among the authorship team to arrive at a set of agreed proposals.

## Results

### Scoping review of the research literature on sustained behaviour change

Thirty distinct candidate behaviour change approaches for achieving sustained behaviour change were identified, listed in Table 1.

**Table 1:** Examples and evidence of how behaviour change has been sustained in settings which could be relevant to embedding COVID‐protective behaviours

Candidate behaviour change approaches for achieving sustained behaviour change	Behaviour change domain
Capability	
Maintain education to maximize knowledge	Hygiene (McMichael & Robinson, 2016), Diet (Wallace, Lo, & Devine, 2016), Diabetes self‐management (StantonFay, Hamilton, Chadwick, Lorencatto, & Gianfrancesco, 2021), GP prescribing (Richards, Toop, & Graham, 2003), Water conservation (Pepper & Brebbia, 2012)
Attempt to build and maintain psychological resources	General (Kwasnicka, Dombrowski, White, & Sniehotta, 2016), Impulsive behaviour (Bosch, Koeter, Stijnen, Verheul, & van den Brink, 2005)
Maintain instruction in action planning	Diabetes self‐management (StantonFay et al., 2021), Weight management (Hoedjes et al., 2017)
Maintain instruction in goal setting	Diabetes self‐management (StantonFay et al., 2021)
Maintain education in how to perform the behaviour	Diabetes self‐management (StantonFay et al., 2021), Neonatal care (Kumar et al., 2008), Weight management (Hoedjes et al., 2017)
Promote sustained self‐monitoring of behaviour	Diabetes self‐management (StantonFay et al., 2021), General (Kwasnicka et al., 2016), GP prescribing (Richards et al., 2003)
Maintain education on how to overcome barriers	Diabetes self‐management (StantonFay et al., 2021), General (Kwasnicka et al., 2016)
Promote rehearsal of the behaviour	Diabetes self‐management (StantonFay et al., 2021)
Maintain demonstrations of the behaviour	Diabetes self‐management (StantonFay et al., 2021)
Educate in experimenting to solve problems	Diabetes self‐management (StantonFay et al., 2021)
Promote pro‐active coping with challenges	Weight management (Thoolen, de Ridder, Bensing, Gorter, & Rutten, 2009), Impulsive behaviour (Bosch et al., 2005)
Train in use of tools or resources	Hygiene (McMichael & Robinson, 2016)
Opportunity	
Provide tools and resources	Hygiene (McMichael & Robinson, 2016), Physical activity (Hafner, Pollard, & Van Stolk, 2020; Mitchell, Lau, White, & Faulkner, 2020), Medication adherence (Free et al., 2013), Smoking cessation (Free et al., 2013)
Maximize usability of tools or resources	Hygiene (McMichael & Robinson, 2016), Product use (Mahamuni, Khambete, & Punekar, 2019), General (Kwasnicka et al., 2016),
Ensure maintainability of tools and resources	Hygiene (McMichael & Robinson, 2016)
Change living, working and travel spaces	Hygiene (McMichael & Robinson, 2016)
Change built environment	Hygiene (McMichael & Robinson, 2016)
Provide continuing leadership	Hygiene (McMichael & Robinson, 2016)
Develop ongoing social support	General (Kwasnicka et al., 2016), Child‐rearing (Gurney‐Smith, Granger, Randle, & Fletcher, 2010), Weight management (Hoedjes et al., 2017)
Attempt to change behavioural norms	Hygiene (McMichael & Robinson, 2016), General (Kwasnicka et al., 2016), Environmental sustainability (Hargreaves, 2011), Child‐rearing (Gurney‐Smith et al., 2010), Water conservation (Pepper & Brebbia, 2012)
Attempt to change social roles	Hygiene (McMichael & Robinson, 2016)
Motivation	
Attempt to shape cultural identity	Hygiene (McMichael & Robinson, 2016)
Attempt to change cultural values	Hygiene (McMichael & Robinson, 2016), Covid transmission (Prosser, Judge, Bolderdijk, Blackwood, & Kurz, 2020)
Promote behavioural goal setting	Weight management (Hoedjes et al., 2017)
Attempt to create sustained emotional responses	
Desirability	General (Kwasnicka et al., 2016), Neonatal care (Kumar et al., 2008), Child‐rearing (Gurney‐Smith et al., 2010), Health behaviours (Petty, Barden, & Wheeler, 2009)
Enjoyment	General (Kwasnicka et al., 2016)
Anxiety	Hygiene (McMichael & Robinson, 2016), Covid transmission (Prosser et al., 2020)
Disgust	Hygiene (McMichael & Robinson, 2016)
Attempt to create sustained sense of personal control	General (Kwasnicka et al., 2016), Hygiene (McMichael & Robinson, 2016)
Maintain financial incentives	Physical activity (Derlyatka et al., 2019; Hafner et al., 2020; Mitchell et al., 2020), Water conservation (Pepper & Brebbia, 2012)
Shape or harness identity or values	General (Kwasnicka et al., 2016), Neonatal care (Kumar et al., 2008)
Attempt to promote co‐ownership of practices	Hygiene (McMichael & Robinson, 2016)
Attempt to change habits	Hygiene (McMichael & Robinson, 2016), Diabetes self‐management (StantonFay et al., 2021), General (Kwasnicka et al., 2016), Active transport (Moser et al., 2018)

Twelve approaches aimed at increasing capability included increasing people’s understanding of what they need to do, how to do it, and why it is important so that it is easy for them to do what is required when it is required (Beard et al., 2019). Building capability will include teaching people how to negotiate social pressure to enter unsafe settings or to behave unsafely in social settings (Beard et al., 2019).

Nine approaches aimed at increasing opportunity focused on providing an environment that allows people to do what is needed when it is needed (Beard et al., 2019). This includes both the physical environment of the spaces they use and the ‘social environment’ of expectations and norms. Developing norms supportive of safer behaviours that substitute for more risky ones will be important (Bavel et al., 2020). While some changes to the physical environment entail large‐scale redesign of public and private spaces that will take place over the medium to long term, some alterations to the ways in which people use space, such as signage about occupancy limits, can be made very rapidly.

Nine motivational approaches are proposed aimed at guiding people to find it more attractive – for whatever reason – to do what is needed rather than not do it, and have the behaviour built into their habits and routines (Beard et al., 2019). Routines may vary in frequency and be regular or context‐dependent, as in if‐then plans, for example, ‘when I enter a crowded indoor space, I will wear a face covering’. Embedding behaviours into everyday life includes generating or tapping into core self‐identities and values that make the behaviours important to people. Social‐identity has been found to be important in how people respond collectively in disaster and emergency situations (Carter, Drury, & Amlôt, 2020) and in helping people to stop smoking (West, Walia, Hyder, Shahab, & Michie, 2010). Fostering social‐identities that value one’s own safety and the safety of one’s community are a powerful means of supporting lasting enactment of Covid protection behaviours. This is exemplified by the ‘we are Scotland’ campaigns, which found that of the almost 60% of adults across Scotland who saw the campaign, 83% agreed that it helped them understand that we are all responsible for keeping Scotland safe. Almost 80% reported taking some action in response with almost half reporting taking more care to use face coverings, distance, avoid crowds, and clean hands or surfaces (Scottish Government, 2020).

### Review of best practice in risk and safety management

We identified nine principles that have been successfully applied to risk and safety management in sectors such as transport, food preparation, and construction and that were deemed relevant to domestic as well as to work settings and public spaces such as in the hospitality and entertainment sectors.

These concerned:

#### People’s understanding of, and response to, risk

The ways in which people understand new phenomena and the risks associated with them are governed by a number of principles. Achieving sustained behaviour change requires taking these into account:

1. People rarely come to their understandings alone or through private contemplation and calculation. Rather, they draw on socially shared understandings that are current in their communities and society. Consequently, different communities may see a phenomenon in different ways. For example, some ethnic minority groups have been historically exploited or neglected by medical authorities, which can lead them to regard vaccination in terms of control rather than public health (Bish, Yardley, Nicoll, & Michie, 2011).
2. Understanding new phenomena is usually anchored by reference to previous phenomena about which people believe they have a clear understanding (Selge & Fischer, 2011). Such anchoring can at times be misleading and lead to dysfunctional responses (e.g., seeing COVID as flu leads to ignoring asymptomatic spread and mixing when one does not have symptoms).
3. A potentially powerful way of embedding representations of new phenomena is through objectification (Devine‐Wright & Devine‐Wright, 2009). This involves using a concrete, easily understood metaphor (Wagner, Elejabarrieta, & Lahnsteiner, 1995). For instance, the process of aerosol spread can be likened to inhaling someone else’s cigarette smoke and hence generate understanding of the contexts where this is likely and the measures necessary to avoid it.
4. Social groups have shared norms for how one should respond to risks (Spears, 2021). Establishing the normative character of risk protection measures, including behaviours such as use of safety checklists and wearing protective clothing, is key to their sustained enactment (Neville, Templeton, Smith, & Louis, 2021; Tunçgenç et al., 2021).

#### Risk management strategies

Achieving successful risk management across a wide range of settings has been found to follow several core principles that align with advice from the SAGE Environmental Modelling Group (UK Government, 2020). Together, these constitute what may be termed an enhanced risk management approach.

1. Employing multiple levels of protection. In organizational safety, a key principle, characterized by the ‘Swiss cheese’ metaphor, involves recognizing that any one layer of protection will allow failures but if one applies multiple layers, each with its own strengths and weaknesses, one can build a more resilient system that minimizes the risk of failure while maximizing the ability to operate effectively (Reason, 2021).
2. Combining physical, social, and psychological measures. Maximizing safety while preserving effective functioning in risky settings involves a combination of creating and providing safe environments and equipment, constructing implementable rules and norms, and providing people with the knowledge, skills, and motivation to make and apply accurate risk assessments alongside the authority and capacity to act in response. Environmental modifications can enable protective behaviours in a way that makes them more likely to happen than relying on people’s knowledge, skills, and motivation (Reason, 2021).
3. Involving all relevant actors. Successful risk management involves ensuring that all key actors are involved: those working in shared spaces, those visiting the spaces, employers, managers, regulators, and those involved in inspections. Participation ensures that management is locally appropriate and ‘owned’ by participants. A useful tool to enable the co‐creation of an effective and appropriate ‘COVID‐secure’ risk management approach is the risk assessment framework (RAF) (which includes a hierarchy of control) (Florin & Parker, 2020; Risk assessment ‐ Working safely during the coronavirus (COVID‐19) pandemic, 2021). The RAF sets out a strategy for prioritizing sharing of information and risks.
4. Effective communication of risk and uncertainty. Behaviours in occupational, health, and other areas of everyday life are strongly influenced by our understandings of and perceptions of risk (Pidgeon, Hood, Jones, Turner, & Gibson, 1992). Two‐way communications with those most directly affected by risks can help characterize current risks, frame and implement practical safety measures, ensure effective uptake of behavioural measures (Arvai, 2013), and identify remaining gaps in risk‐reducing and safety‐enhancing measures. In developing risk communications for behavioural measures, it is important to characterize and take account of people’s existing beliefs or ‘mental models’ about the risk, and address misunderstandings or key knowledge gaps (Morgan, Fischhoff, Bostrom, & Atman, 2002). Communications also need to be fully pre‐tested for understanding and acceptability before implementation (Pidgeon & Fischhoff, 2011) while trust in others is an important factor influencing both perceptions of risk and acceptance of protective measures.
5. Continued monitoring of risk levels and adjustment of protective measures according to those levels. This involves setting expectations about the implementation of measures, monitoring whether these expectations are achieved and if not, amending practice to improve implementation of measures. Research has shown that, if risks are complex and changing or information is in part uncertain, staying safe involves proactive reflection on risks, ongoing evaluation of existing safety measures, and adjustments where necessary (Pidgeon, 2021; Sutcliffe, 2006). This will in turn require action by multiple types of actors, including both people and organizations.

### Review of reports from the UK Government’s behavioural subgroup of the Scientific Advisory Group on Emergencies (SPI‐B)

SPI‐B emphasized that information needs to be provided at two levels. One is to do with an overall understanding of the pandemic and of the processes of infection transmission (mental models and social representations). The other is to do with the identification of hazards and mitigations where clarity and specificity are critical, and people need both to know what to do and to be able to do it.

When considering strategies for sustaining adherence to infection control behaviours, SPI‐B recommends providing positive feedback on behaviours; emphasizing that everyone has a role to play; promoting positive alternatives to restricted activities; helping people change their environments, and to identify risky situations; focusing on reducing infection risk rather than compliance; and providing targeted information and practical support and adequate resources to enable all sections of the community to do what is asked of them such as self‐isolation. Failure to provide support both lowers adherence and exacerbates inequalities (Derlyatka, Fomenko, Eck, Khmelev, & Elliott, 2019).

SPI‐B also noted that there are emotional barriers to social distancing and mask wearing that may interfere with personal interactions (SAGE, 2021). Interventions need to be co‐designed with families and communities to create acceptable strategies for reducing risk while avoiding excessive burden and maintaining family and community cohesion (Moser, Blumer, & Hille, 2018; SPI‐B, 2020b). There is more likely to be a positive response to interventions if the reasons behind changes are fully explained and understood. Clear communications are required to avoid interventions being seen as arbitrary or discriminatory. Communications should emphasize care rather than punishment and be culturally appropriate. They should be co‐created and delivered with trusted community voices to maximize engagement and make it clear that interventions are for the benefit of, and endorsed by, the community, rather than the result of external enforcement (Beard et al., 2019).

When considering workplace infection control, SPI‐B emphasized the importance of co‐creation in designing layers of protection (as opposed to single solutions). Co‐creation requires full consultation with all key stakeholders (Martin, Hulland, Dreibelbis, Sultana, & Winch, 2018). It also noted the importance of clear risk communication to empower individuals to protect themselves and colleagues and to be vigilant at all times, including during breaks in work (risks from social interaction in staff rooms were noted) and commuting. Engagement and education will be needed for both relevant occupations and the general public (McEwen, Preston, & West, 2002).

There is evidence that a participative approach to identifying physical, environmental, and behavioural changes needed is more effective (Bonell et al., 2018) because: (1) those within a setting are best placed to make sensible decisions about it, and (2) people are more likely to support changes if they have been involved in shaping them.

SAGE noted the benefits of the Community Champions programmes in identifying and facilitating context‐specific solutions and in reaching isolated or marginalized groups to communicate health messages and offer support (Derlyatka et al., 2019; Gillespie & Marshall, 2015; UK Government, 2020b). They are likely to be especially effective in contexts where trust in government is low and where community engagement is required to build trust, address disinformation, and ensure interventions are appropriate to local contexts. To achieve this, Community Champions need autonomy to secure participation and identify activities that meet the needs of particular communities, and sustained resourcing and financial and practical support (e.g., access to settings, equipment).

### Development and review of proposals

The literature review revealed that the direct evidence base on how to effect long‐term behaviour change needed to sustain lower transmission of SARS‐CoV‐2 is relatively sparse (though improving). Further research is needed to understand people’s levels of understanding and mental models of Covid‐protective behaviours, the barriers to change and the most effective approaches to overcoming them. Nevertheless, the extensive literature on broader public health and behavioural science is relevant and useful in developing strategies for achieving the desired behaviour change.

Through discussion of the literature reviewed, we identified ten strategies for achieving infection control, categorized them according to targeting Capability, Opportunity and Motivation, and identified examples of how these can be applied in relation to infection‐control behaviours (Table 2). In summary, these involve building Covid‐safe educational programmes, regulating to ensure minimum standards of safety infrastructure in public spaces and workspaces, using communications and social marketing to develop a Covid‐safe culture and identity, and providing resources so that all sections of society can build Covid‐safe behaviours into their daily routines.

**Table 2:** Strategies for ongoing infection control

Target	Examples of strategies
Capability	Build and sustain an understanding of infection risks and how to mitigate these through: 1. Multichannel information and comms campaigns, including in schools, workplaces, venues to explain why, e.g., outdoors vs indoors or face coverings can reduce transmission. e.g., education and training in self‐management has proved effective in achieving lasting improvements in diabetes self‐management (StantonFay et al., 2021). e.g., informational campaigns have been found to be an important part of cost‐effective interventions to a range of improve health‐related behaviours (Beard et al., 2019). 2. Education on infection risk management across educational settings from schools to HE and professional training. e.g., training in use of resources has proved effective in sustained improvement in hygiene behaviours in low income countries (Martin et al., 2018). e.g., continued education and training has been found to support sustained changes in GP prescribing patterns (Richards et al., 2003). 3. Providing resources that are easily accessible and usable by all members of the community. e.g., simple post‐it type pad for GPs to keep on their desks led to an increase in delivery of advice on smoking (McEwen et al., 2002). e.g., checklists and templates developed to promote safe practice in surgery (Gillespie & Marshall, 2015).
Opportunity	Ensure that all sectors of society and organizations work together to maximize opportunities for successful risk management by: 4. Providing practical, regulatory, and financial support for the creation of home, work, leisure, and transport environments that enable adequate physical distancing, ventilation, and wearing of face coverings when the need arises. e.g., website with accessible information about ventilation status and opportunities as implemented by New York City Department of Education (NYC Department of Education, 2021). e.g., Government providing guidance for tenants, landlords, and local authorities to reduce in‐household transmission. Local authorities may be able to use their enforcement powers in relation to landlords to deal with a serious overcrowding hazard (UK Government, 2021). 5. Provide practical support and resources to ensure the sustainability of Mutual Aid groups, which provide multiple forms of practical support to those in the community, from delivering food, providing emotional support, to walking the dog (Fernandes‐Jesus et al., 2021). 6. Ensure people have sufficient and sustained financial and other resources, including employment protection, to be able to behave in ways that mitigate risks. e.g., ensuring that there is adequate financial and material support during a period of self‐isolation or quarantine (Webster et al., 2020). 7. Building strong social norms around infection control behaviours such as physical distancing and mask wearing of the kind seen in some other countries. e.g., effect of shaping social norms on a range of Covid‐protective behaviours and environmental sustainability (Hargreaves, 2011; Neville et al., 2021; Pepper & Brebbia, 2012). e.g., formally engaging community leaders in a programme to achieve lasting changes in health‐related behaviours (Yajima, Takano, Nakamura, & Watanabe, 2001).
Motivation	Ensure that people and organizations attach high value to infection control and how this is embedded into daily lives by: 8. Using all available communication channels to strengthen social‐identities, values, and emotional responses around infection prevention and mitigation, and a sense of personal control. e.g., large effect of a programme targeting emotional drivers of hand‐washing with soap (Biran et al., 2014). 9. Specific community engagement initiatives with minorities and marginalized social groups e.g., Scottish NHS co‐production initiative for community health and UK’s community champions scheme (Loeffler & Power, 2013; UK Government, 2021). 10. Providing training and resources to build habits and routines into people’s lives, for example taking a face covering with you when leaving the home or opening a window when someone visits. e.g., regular prime‐time TV segment based on behavioural science principles (Veiligheid, 2016). e.g., habit building has proved effective across a range of health‐related behaviours (Beard et al., 2019).

In all reviews, there was evidence that minority and socio‐economically deprived groups face several barriers in applying risk‐mitigating practices in their workplaces, communities, transport, and domestic spaces (UK Government, 2021). These barriers have contributed to a higher age‐standardized mortality rate in the first and second wave (Nafilyan et al., 2021). Examples of barriers are:

1. *In workplaces,* less capacity to negotiate workplace safety due to precariousness of work, less ability to counteract instructions from managers, or inherently poor quality of the environment (Moyce & Schenker, 2018; SPI‐B, 2021; The vulnerable worker in Britain & problems at work ‐ Anna Pollert, Andy Charlwood, 2009).
2. *In communities,* greater reliance on informal social support networks for care for children, the elderly, and unwell, means that community members, particularly women, may face greater exposure. These are moral relationships and intimate situations where it may be difficult to enable protective behaviours unless targeted advice is given on negotiating these (SPI‐B, 2020a).
3. *In domestic spaces*, multigenerational households or houses of multiple occupation are environments, where due to poor housing stock and density it is very difficult to self‐isolate or maintain physical distancing (Housing, Household Transmission, & Ethnicity, 2020).

Tackling these barriers may require additional, targeted measures, although we found limited evidence as to what works. Organizational‐level and settings‐based interventions (e.g., covid‐safe workplace practices) may be less likely to generate inequalities than individual‐based interventions because they are less dependent on individual choices and actions. Past advice from SAGE’s ethnicity subgroup and SPI‐B addresses the complexity of this area and the potential for unintended stigmatization of particular groups (UK Government, 2020, 2021; UK Government, 2020). Any risk mitigation interventions or communications should avoid stigmatizing particular communities, regions, or groups (SAGE, 2021). Stigma contributes to disengagement from health‐protective behaviours and can directly contribute to perverse, negative health‐effects (Hatzenbuehler, Phelan, & Link, 2013). On the other hand, greater social cohesion has been found to generate more trust in Covid‐19 government measures, which leads to greater adherence to health measures and therefore increased engagement with risk mitigation (Lalot, Abrams, Broadwood, Hayon, & Platts‐Dunn, 2021). Stigma is likely to be mitigated by adequate co‐production and pilot testing with affected communities.

Finally, the self‐organization of the community in mutual aid groups has played a crucial role in providing support and services that were beyond the capacity of national and local government and third sector organizations (Curtin, Rendall, Roy, & Teasdale, 2021). However, it is very difficult for such groups to sustain themselves over an extended period of time. The Government can play an important part in scaffolding such self‐organization by, for instance, providing resources and providing payment for volunteers (Fernandes‐Jesus et al., 2021). There are successful examples of this in the UK’s Ministry of Housing, Communities and Local Government (MHCLG) Community Champions scheme that has operated through two mechanisms. The first via decentralized funding to local authorities, which has then been devolved to third sector and micro‐community organizations. They have drawn on micro‐knowledge, cross‐group coordination, and awareness of barriers to produce a greater uptake of vaccination among disadvantaged and minority groups. The second, via large national voluntary sector partners, Near Neighbours and Strengthening Faith Institutions, who have reached micro‐communities that they have worked with over the longer term (Bear, 2021).

## Discussion

Successful risk management and sustaining behaviour change involves: multiple layers of protection; a combination of physical, social and psychological measures; effective communication of risk and uncertainty; inclusion of the targeted groups in its development; and continued monitoring and feedback. Interventions aiming to achieve long‐term behaviour change need to consider how people understand new phenomena and the risks associated with them, including the differences in understanding and perception between communities, the role of anchoring to previous similar phenomena, and how the use of concrete metaphors can foster understanding.

The strategies are all directed at making the desired behaviour Normal, Easy, Attractive, and Routine (NEAR) (West et al., 2020) by increasing multiple levels of capability, opportunity and motivation. The level of support and changes required to achieve this will differ depending on the cost or burden associated with particular behaviours; some behaviours (e.g., working from home) may take more to make them NEAR than other behaviours (e.g., hand washing).

Minority and socio‐economically deprived groups face major barriers in applying risk‐mitigating practices in their workplaces, communities, transport, and domestic spaces, as is clear from the unequal mortality rates they have experienced (Government, 2021). Additional measures aimed at overcoming these barriers are required but need to avoid stigmatizing the groups concerned. This is best achieved by interventions that create environments to avoid or overcome barriers, complemented with targeted, co‐produced communication interventions.

The need for a multi‐layered, multifaceted approach to long‐term behaviour change requires the co‐ordinated participation of an array of public and private sector organizations rather than a series of separate interventions. Governance of the design and implementation of policies is important in achieving this: each would benefit from being supported by technical expertise, a logic model, co‐production between internal and external stakeholders and a scientific evaluation plan.

Tracking of adaptation to change should be used to guide decision‐making in an ongoing, iterative manner before, during, and after implementation, on potential negative as well as positive outcomes. Methods for gathering data include qualitative research, direct observation, routinely collected organizational metrics, randomized trials, natural experiments (with non‐random comparators), and time series studies. Co‐production and extensive stakeholder engagement will be critical to the success of monitoring and research, as well as interventions.

The bringing together of literature on maintaining behaviour change, on the social psychology of group influence and on managing risk in infection control and other domains, together with relevant reports produced by SPI‐B has identified a wealth of strategies that are likely to be helpful in enabling long‐term behavioural changes to control Covid‐19 and hence minimize the chance of future waves and lockdowns, and to prevent or minimize harmful impacts of future pandemics. The rapid nature of these review activities, conducted in response to a policy question designed to inform the ongoing response to a major public health emergency, may have resulted in some key sources of insight being missed. The inclusion of a review of previous SPI‐B papers may have helped mitigate this limitation as a number of these papers were informed by rapid systematic reviews, and consultation with subject matter experts. Expediency will always be necessary in an evolving crisis (Rubin, Wessely, Greenberg, & Brooks, 2021), but continuing efforts should be made to inform the recommendations presented here.

The relative importance of the strategies outlined in this paper will also depend on the trajectory of the Covid‐19 pandemic, and whether endemicity or elimination is the likely ‘end state’. Whilst the recommendations we have set out apply to ongoing Covid‐19 control strategies, they apply equally to efforts to improve infection prevention and control practices in community and healthcare settings for a range of infectious disease threats (Klinke, Renn, & Goble, 2021).

This paper has stressed some of the generic principles of risk management and resilience obtained from longstanding research into the ways that organizations faced with uncertain risks deal with them to deliver safety. The conclusions and recommendations from that research evidence clearly apply most directly at the organizational level of action when thinking about promoting Covid‐safe behaviours – that is for organizing the operation of the many entities (workplace, retail, leisure, transport etc.) which hold a responsibility for influencing the safe behaviours of employees and users for whom they assume responsibility. While individual behaviour is always a key component of such organizational resilience, collective risk management principles need also to be be applied. This paper suggests how we can apply these principles to households and members of the larger community but clearly more work is needed to validate our suggestions.

### Conclusions

Embedding ‘Covid‐safe’ behaviours into people’s everyday routines will require a concerted programme to shape the financial, physical, and social infrastructure. Education, regulation, communications and social marketing, and provision of resources will be required to ensure that all sections of society have the capability, opportunity, and motivation to enact the behaviours long term. The behaviours that are prioritized and how they are enabled should be part of a society‐wide conversation, informed by epidemiological evidence as to which behaviours are likely to have the most impact in different situations. Such long‐term changes are critically dependent upon changes in the financial physical and social infrastructure at a societal level, across all places where people mix (Bear et al., 2020). Only if this happens, will people be in a position to act in ways that keep themselves, their families and their communities safe.

## Conflicts of interest

All authors declare no conflict of interest.

## Author Contribution

**Susan Michie:** Conceptualization (equal); Formal analysis (equal); Methodology (equal); Writing – original draft (equal); Writing – review & editing (equal). **Robert West:** Conceptualization (equal); Formal analysis (equal); Investigation (equal); Methodology (equal); Writing – original draft (equal); Writing – review & editing (equal). **Nick Pidgeon:** Conceptualization (equal); Investigation (equal); Methodology (equal); Writing – review & editing (equal). **Stephen Reicher:** Conceptualization (equal); Methodology (equal); Writing – review & editing (equal). **Richard Amlȏt:** Conceptualization (equal); Methodology (equal); Writing – review & editing (equal). **Laura Bear:** Conceptualization (equal); Methodology (equal); Writing – review & editing (equal).

## Data Availability

Data sharing is not applicable to this article as no new data were created or analysed in this study.
